# Supplementary material for: Increased error-correction leads to both higher levels of variability and adaptation
Source: PLoS One. 2020 Feb 4;15(2):e0227913. doi: 10.1371/journal.pone.0227913 (PMC6999875; doi:10.1371/journal.pone.0227913)
Supplement: Attachment — Submitted filename: Response to Reviewers.pdf [file pone.0227913.s001.pdf]

Response to the reviewers

The authors would like to thank the reviewers for their insightful comments on our manuscript. We have carefully evaluated each of their responses and concerns. We also thank the reviewers and editor for their patience and extending the deadline for our re-submission. Each of the comments will be evaluated separately below.

Reviewer 1

The authors investigated how movement variability is related to motor adaptation in the temporal domain. Although the motivation of the study and the target-intersection task sound interesting, I cannot agree with the validity of the analysis in the current paper. Please respond to the following questions carefully. In sum, I was interested in the target-intersecting task, but I cannot be favorable for almost all the results.

Dear reviewer 1,

Thank you very much for your evaluation. The points that were brought up have proven very useful to us when improving our manuscript. We hope that we have clearly understood your concerns and that you are satisfied with our answers and corrections.

1. Even under assuming straight pen trajectories, this task is redundant. That is, subjects can choose the pair of initial movement angle and

movement velocity to achieve the task. The authors should investigate how the pair is modified depending on perturbation and task set; however, they investigated only movement velocity. These missing analyses can hide significant findings inherent in this interesting experiment. Without the analysis of the movement angle, it is not worth to discuss their results. We acknowledge that interception location plays a role when evaluating the movements of our participants. Variability in movement speed and aiming angle are correlated in our data (see figure 1 below). The reason why only movement speed was analyzed was because of the hypothesis that was the base of the research. As previous research had claimed exploration (and as a result variability) could benefit error-based learning, our hypothesis followed this same rhetoric. We assumed that theoretically it would make more sense that exploration of movement speed would benefit temporal adaptation over exploration in the movement angle, (and movement angle might benefit spatial adaptation more). However, we realize that this assumption might blind us from other behavior that could have benefited adaptation. In order to get more information on this idea we have modeled Partial Least Squares Regression (PLS) on the adaptation scores with absolute movement angle variability σ_{Ma} and movement speed variability σ_{Mv} as predictors. This PLS can give us information on the

contribution of different correlated predictors on a dependent variable.

$$AdaptationScore = 1.27 \cdot \sigma_{Mv} + 0.56 \cdot \sigma_{Ma} - 1.33 \cdot (\sigma_{Mv} \cdot \sigma_{Ma})$$

This relationship in itself is very interesting, because together a larger part of the Adaptation Score can be explained than by just the σ_{Mv}. It indicates that both σ_{Mv} and σ_{Ma} have a positive main effect on the Adaptation Score, but that either high or low values in both negatively affects this relationship. However, of these predictors, only for σ_{Mv} the estimated (bootstrapped) 95% confidence intervals that are different from zero (see figure 2), indicating that only σ_{Mv} has a significant effect on the Adaptation Score. We therefore believe that our initial assumption, in which we stated that specifically movement speed is beneficial for temporal adaptation, is not violated. However, we understand that other readers might have these same questions, which is why we have added the results of the above mentioned PLS to the manuscript and discussed its implications further in the discussion section.

2. In the adaptation, we can assume a simple state-space model,

$$Mv(t+1) = \lambda * Mv(t) + \eta * (TE(t) + \zeta(t)) + xi(t)$$

where lambda is forgetting rate, eta is learning rate, xi(t) is motor noise, and zeta(t) is sensory noise to perceive TE(t). Under this equation, it

is self-evident that the larger σ_{Mv} results in faster learning, because a high magnitude of $xi(t) + eta * zeta(t)$ (i.e., σ_{Mv}) is nearly equivalent to high learning rate. Please mention clearly that the current results are not mere results of high sensory noise.

One important way in which adaptation can be modeled is with a state space model. Another way it can be modelled is with a Bayesian tool, for example a Kalman filter. The reason that these Bayesian of models are used is that learning from errors is often thought to be optimal. More uncertain feedback (due to sensory and/or motor noise) leads to smaller error updates. Although higher sensory noise generally leads to more variability in movement outcome, we would not expect learning rate to be higher because of it. Furthermore, assuming that zeta would be gaussian noise with mean of zero, we are not sure if the error would always be perceived as larger and therefore updates would be larger. The measure of error (TE) in our task is 1 dimensional. This means that the noise could have decreased the perceived error just as likely as it could have increased it, which means on average increased correction of the error is not expected. We have extended the text about noise in the discussion a bit to also cover sensory noise.

3. I wonder how generalization affected the results. Because the authors used three different speeds for target motions, they need to coednsider how the adaptation in one speed can be generalized to other speeds. Another possibility was the participants with small σ_{Mv} showed little

generalization compared to those with large σ_{Mv}.

The three different speeds were used to prevent participants from learning were to intercept at what time, without taking the movement of the target into account. Given the size of the dataset, we cannot be sure if the different speeds have an effect on the results. Some participants might have larger generalization than others (with the term generalization referring to how the mapping between action and consequence generalizes to other tasks/speeds). It is possible that at the moment the perturbation was applied, the broadly generalizing participants were able to generalize the changing consequences better for all target speeds and adapt faster as a result. However, higher generalization would not directly lead to more variable movement speed during the baseline.

On the other hand, increased movement speed variability might in itself lead to more generalization (as proposed by the reviewer's second suggestion). Hence, more variable people might develop broader tuning curves, and as a result adapt faster. We think this idea is captured well within the notion of *exploration* that is part of our original hypothesis. Research has shown that generalisation temporal perturbations is limited only to tasks that are very similar to the learned task (de la Malla et al, 2014). Higher variability due to exploration might broaden the brains knowledge on the temporal contingencies over a broader range of target speeds and situations. Unfortunately our current dataset can-

not give a clear answer to the question of generalization. As discussed in manuscript, we suspect the variability is a result of error correction rather than exploration. However, we understand that others might have the same question and have therefore dedicated a section to it in the discussion.

4. Please discuss slope and asymptote separately. Why did the authors sum those values to quantify Adaptation Score?

   Adaptation is often calculated by fitting an exponential function on the data and calculating the Time Constant (TC) of this best-fit. A smaller TC indicates better adaptation. This method assumes that adaptation takes place according to an exponential function. We aimed at another way of calculating adaptation, as during pilot studies we were not able to calculate this fit for each participant, indicating that not each participants adaptation can be quantified this way (See figure 3). We opted for an Adaptation Score (AS) that could be determined for each participant so that we could also account for the participants that had less typical types of adaptation, for example more linear. The reason we normalised and summed the two in order to calculate the AS is because both slope and asymptote determine the quality of the adaptation. Very low levels of adaptation (asymptote, b) might be achieved with a slope (a) that is steep, while a higher level of adaptation might be achieved with a low slope. It is the participants that have both fast (slope) and high (asymptote) adaptation that have high AS,

while slow and low adaptation would lead to low AS. Although we are unsure if this is the best way of quantifying the adaptation, we have found that (for the participants we were able to calculate the TC of the exponential for; n=11), the TC was negatively correlated with the AS (albeit not significantly; R = - 0.54; p = 0.088). This indicates that the AS can replace TC as a measure of adaptation. Figure 4 below shows how the AS and TC are related. When we use the TC instead of the AS to correlate the relationship between $\sigma_M v$ and adaptation, we find a significantly negative correlation (R = -0.63, p = 0.038), providing more evidence that our measure of adaptation yields similar results to the more standard ones. We have added these results to the manuscript.

5. Despite the clarity of Fig. 3B (if the Adaptation Score were a valid value to quantify adaptation), Figs. 4-6 do not seem meaninful. The reason is the small correlation in Fig.3, cross-correlation that does not seem to be significantly different from 0 in Fig. 4, and low correlations in Fig.5. In Figs. 3 and 5, it seems impossible to predict one variable based on the other. We acknowledge that we have not reached a defining answer to the question of what the reason behind the variability-adaptation relationship is. The data hints towards increased variability being due to the use of movement speed in order to correct for error. We aimed to convey the idea that the results regarding the cause of the relationship are non-conclusive, however we might not have fully

succeeded. We will state more clearly that further research is needed to find more conclusive answers.

References:

de la Malla, C., Lpez-Moliner, J., & Brenner, E. (2014). Dealing with delays does not transfer across sensorimotor tasks. Journal of vision, 14(12), 8-8.

Reviewer 2

The authors examine the relationship between variability in hand movement speed during baseline and the adaptation to a temporal perturbation of sensory feedback (delay). They find that baseline variability is a good predictor of adaptation expressed as a summary score including the rate at asymptote level of adaptation. The authors argue that the increased baseline variability seen in good adapters is not due to exploration, but rather to increased correction of errors in previous trials.

The source and relevance of motor variability, and the explanation of individual differences in motor learning, are critical issues in the field of motor control and learning. The authors provide important novel insights into the role of exploration and variability in motor learning. The experiment is well designed, and the paper is generally well written and concise. Dear reviewer 2, thank you for your kind words and your suggestions. We found them very valuable when rewriting the manuscript. We hope to have answered your questions and concerns to your satisfaction.

1. My main comment is that the analyses of the relation between variability and adaptation should be more clearly explained and motivated. How/why were the particular analyses performed? Why was the adaptation score calculated this way? What is meant by sequential effects? How were the participants divided into groups? Why was the linear model fitted to the group data, and the cross-correlation performed per participant? Why are both a Pearson and a Spearman correlation reported; how exactly are variability and adaptation related? Why do some analyses test differences between groups, while other analyses focus on correlation of baseline temporal variability with other measures? We have expanded the methods section to more clearly to account for the uncertainties and questions of the reviewer. In summary, we have added the use of an exponential function, more widely used in adaptation, and found a correlation with our score (see Fig 3 and 4). Once we have shown this correlation, we keep our score because, unlike the exponential function, we were able to fit our score to all subjects. Concerning the use of the ccf, it only makes sense to apply to individual data, since averaging would factor out the individual corrective patters. This analysis has been done in the spatial domain (see van Beers, 2009). While the lmm can capture individual variability in the random structure of the model. We used Spearman, in addition to Pearson, because Spearman (based on ranks) captures monotonic relation better than Pearson (which requires linear relations). We have rewritten the

methods to motivate our choices more clearly.

2. Another important point is that the term exploration should be clearly defined. Is this an actively controlled (or deliberate) process, or an automatic, implicit process? The term exploration strategies (used by the authors) makes me think that it is a deliberate process, but I am not sure this is what the authors mean. This part could indeed use some clarification. The process is thought to be actively controlled. We have rewritten the section (note that Q6 is incorporated in this section as well)

3. Lines 4-18. I understand what the authors are saying here but the reasoning is a little confusing. They first argue that we need sensory feedback, and then say that sensory feedback is too slow for corrections and we thus need an internal model. It might be helpful to distinguish between immediate corrections and adjustment to changes. In addition, we also need to predict the trajectory of the ball if were intercepting a moving object and rely on sensory feedback. Please clarify this paragraph. Agreed: we have rewritten the paragraph.

4. L 22. neural variability. If the authors refer to execution noise as well as planning noise, please remove neural. Corrected

5. L 30-31. In situations in which error feedback is not clear I think not available would be the correct term here. If not, please explain what is meant by not clear. Corrected to "unavailable or uninformative"

6. L 33-35. It can also be beneficial . in the explored dimension of the behaviour. Its not clear to me what this means, please expand. Changed in order to increase clarity.

7. L 36-38. Since the Wu et al paper is a main motivation for the current study, I think it would be helpful to expand the description of their findings a little bit. Expanded

8. L 42-45. It is not clear to me how exploration strategies could directly benefit the updating of the forward model, please clarify. And what is the potential role of variability from other sources than exploration? Exploration strategies can help us learn about the outcome of our motor commands. When we explore more, we get richer information about the relationship between the motor commands and its sensory outcome. We have expanded our explanation of exploration. We also added some more information about other forms of noise, like sensory noise.

9. Fig 1B. Display 4 is missing a title. It is unclear what the figure below the 4 displays belongs to. Corrected

10. L 92-93. What was the size of the target and the cursor? Added

11. L 109-110. 0.5 cm from where? Added

12. Fig 2 - legend. The word summarized should be summed. Corrected

13. Were the results collapsed for the different target speeds? We indeed did not look at the differences in target speed. Target speed was one

of 3 speeds leftwards or rightwards random. We chose different target speeds so that the participants could not just learn a specific location and time to intercept at each trial. However, our dataset is not large enough to do the analysis for different. However, in accordance with the comments of reviewer 1 (Q3), we elaborated about the use of the different speeds and its affect on generalization in the discussion.

14. L 177. There seems to be a slightly faster adaptation for the HIGH group than for the LOW group. This is not clear to me from figure 3A. Was the slope or asymptote level of adaptation different between groups? This indeed was initially only visual inspection and was after verified with the Adaptation score. We rewrote this section.

15. Fig 4. I would just like to note that I dont find this figure particularly convincing. From the scatter, it looks like there is no relationship in either of the groups. We acknowledge that we have not reached a defining answer to the question of what the reason behind the variability-adaptation relationship is. The data hints towards increased variability being due to the use of movement speed in order to correct for error. We aimed to convey the idea that the results regarding the cause of the relationship are exploratory, however we might not have fully succeeded. We will state more clearly that the data of the second part is exploratory and should be aimed to be reproduced in future studies.

16. L 206-207. Shouldnt the lag zero correlation be positive for both

groups? Lag zero cross correlation was more positive in the HIGH variability group. The HIGH variability group moved in more variable speeds and it might be that the relationship between movement speed and error might therefore more easily revealed. It could also mean that the HIGH variability group had a stronger relationship between movement speed and error, they were more likely to use movement speed as a correction mechanism in future. We expanded this part in the discussion a bit further.

17. Fig 5. What do the error bars represent? Error bars represent the 95% bootstrapped confidence intervals. We have now added this in the figure description as well.

18. L 213-214. A word is missing in this sentence. Corrected

19. Could the authors speculate about the origin of differences in error correction? For example, do people have differences in error sensitivity? Is the mechanism similar to differences in adaptation rate resulting from differences in error size or different levels of uncertainty of sensory feedback? One of the mentioned mechanisms in the Discussion is a possible difference in planning noise, which might lead to more uncertain predicted sensory feedback (hence more correction of error) or enhance the detection of errors. We expanded a bit on that topic and added the role of sensory noise. We also included a part about a possible role of generalization on error correction and the idea that error can be

interpreted as having a more spatial or more temporal origin.

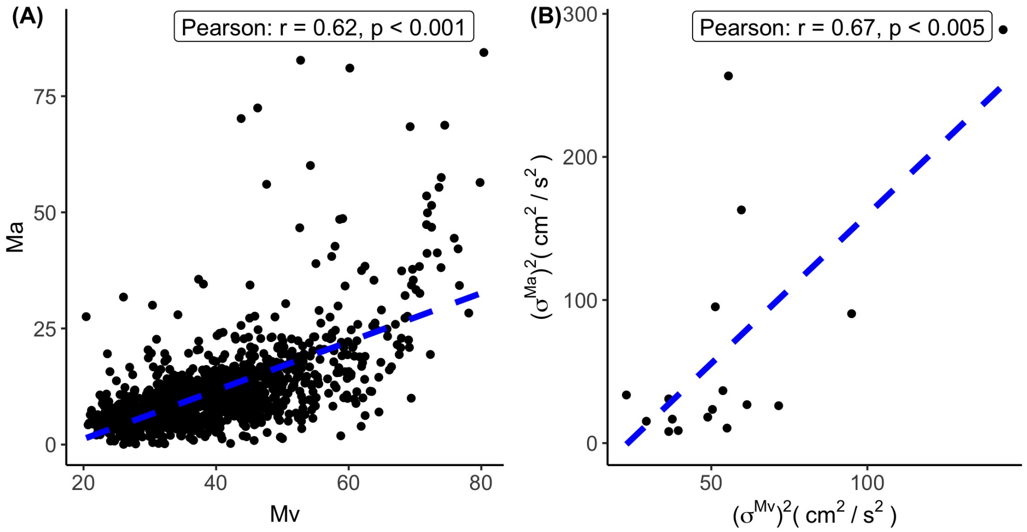

Figure 1: A. Correlation between movement speed and movement angle. The dots represent the individual trials. B. Correlation between the variability in movement speed and the variability in movement angle. The dots represent the individual subjects.

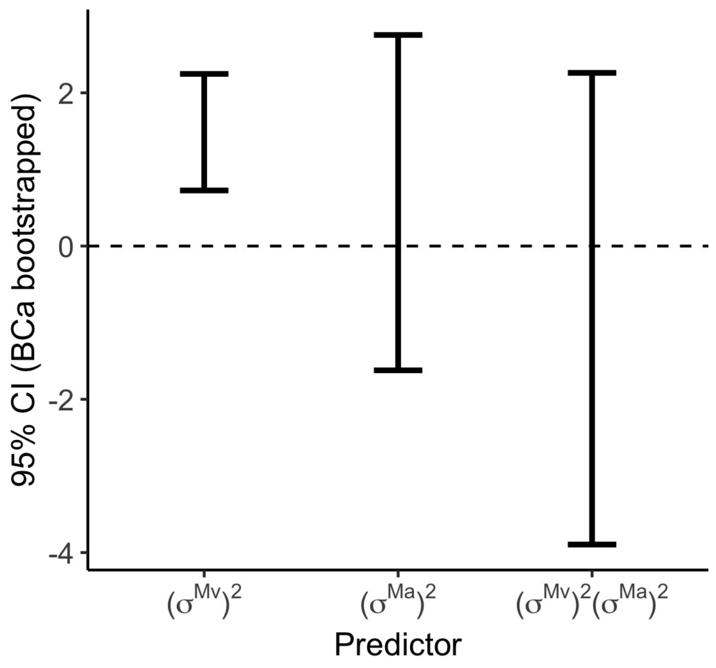

Figure 2: The BCa bootstrapped confidence intervals for each of the predictors.

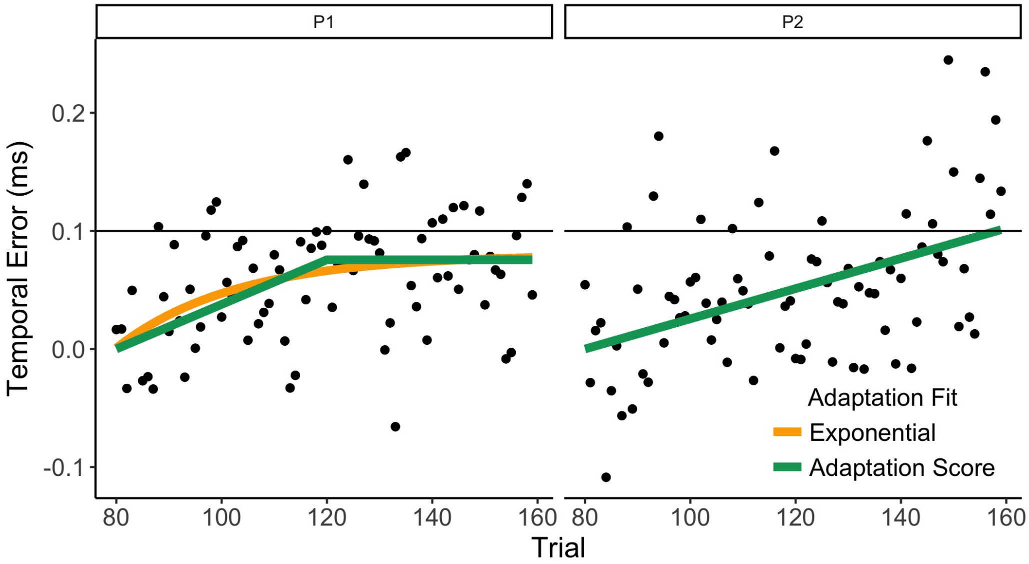

Figure 3: The adaptation fit through and exponential fit (yellow) and our Adaptation Score (green). For P2 it was not possible to fit the exponential on the data.

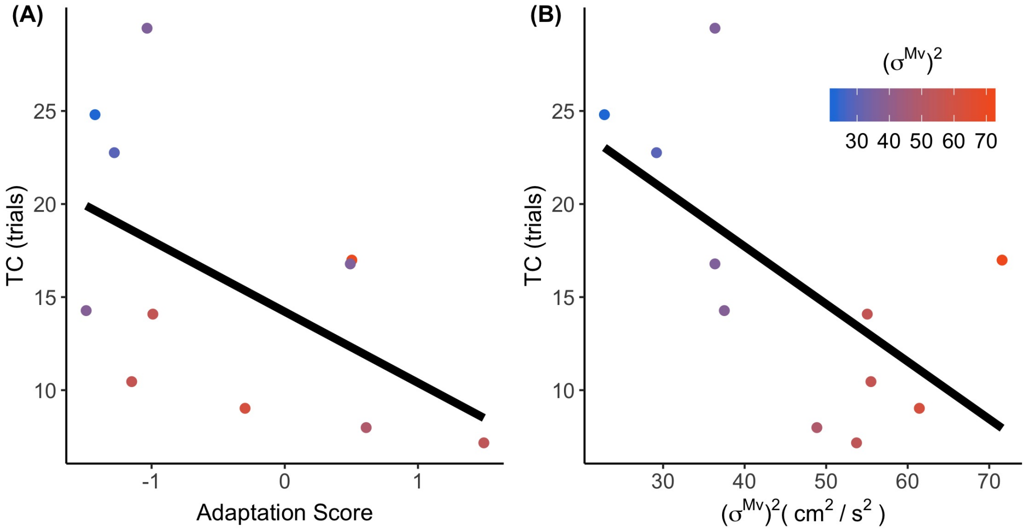

Figure 4: A. The relationship between the Adaptation Score and the time constant of the exponential function. B. The relation between movement speed variability and the time constant of the exponential function.
