# Supplementary material for: Increased error-correction leads to both higher levels of variability and adaptation
Source: PLoS One. 2020 Feb 4;15(2):e0227913. doi: 10.1371/journal.pone.0227913 (PMC6999875; doi:10.1371/journal.pone.0227913)
Supplement: Attachment — Submitted filename: Response_Reviewers.pdf [file pone.0227913.s002.pdf]

Dear Editor,

Thank you very much for your work and the chance to resubmit. We believe we have addressed the points reviewers' point.

Review Comments to the Author

Please use the space provided to explain your answers to the questions above. You may also include additional comments for the author, including concerns about dual publication, research ethics, or publication ethics. (Please upload your review as an attachment if it exceeds 20,000 characters)

Reviewer #1: The authors revised their manuscript carefully. Although they missed discussing the effects of generalization, their citing paper can support to interpret the effects of generalization to some extent (but not wholly, of course). I have now a positive attitude towards the acceptance of this manuscript after responding to the following comments. I have two more comments about the PLS, the evaluation of the adaptation score, and discussion.
Dear reviewer, thank you for your comments. Below you can find the answers to other points raised. We hope we have sufficiently satisfied your concerns.

1) Although I understand that they want to focus on the adaptation score, the temporal error (TE) is a more direct measure of adaptation without any post-hoc calculation of such as variability. Although I understand the difficulty of calculating the relation among TE, movement angle (ma), and movement velocity (mv), recent studies enable us to evaluate the relation using a data-driven technique [1]. Further, it is possible to calculate the task-relevant and task-irrelevant variabilities with the technique. It should be better to discuss the relation between task-relevant variability and adaptation score after clarifying the relation among TE, ma, and mv.In the analysis of PLS, they considered only a simple second-order interaction of ma and mv. I guess that the TE can be affected by mv*T*sin(ma) under the assumption of the constant velocity through the movement time T (although this assumption is probably wrong). It should be investigated the more appropriate relation between adaptation score and some kinematic variables.

We agree with the suggestion to check the relationship between different kinematic variables and TE. We used a LASSO regression to analyse explanatory predictors (similar to the Ridge regression in the suggested literature). This showed us that the movement time was one of the main predictors of Temporal error. We already saw that To not exhaust the reader with too many different types of analyses we have also changed the PLS to a LASSO regression to see the relationship between sigma_mv, sigma_ma and adaptation.

2) In l.72-76, they mentioned that the history of error of future error could affect adaptation. Why not consider the influence of predicted error on motor adaptation [2]? Because we cannot be sure what kind of information affects motor adaptation, we should keep our scope broad.
We agree with the reviewers comment and have added some information about prospective errors in the introduction.

ref:
[1] Furuki D & Takiyama K, 2019, Decomposing motion that changes over time into task-relevant and task-irrelevant components in a data-driven manner: application to motor adaptation in whole-body movements, Sci Rep
[2] Takiyama K, Hirashima M, Nozaki D, 2015, Prospective errors determine motor learning, Nat Comm

Reviewer #2: I am happy with most of the changes that the authors made. However, I do have a few more comments, as outlined below. Line numbers refer to the manuscript with tracked changes.
Dear reviewer, thank you very much for your further comments. We hope we have satisfied your final notes.

Line 195-197. "The slope (a) and asymptote (b) that resulted in a minimum residuals were first normalized across subjects and then summed." I realized that the question that I asked in the previous round, "Why was the adaptation score calculated this way?", was rather vague. I am happy that the authors clarified why they used this score rather than the time constant of an exponential function. However, I also meant to ask whether the authors expected variability to influence both the slope and the asymptote of adaptation? What was the reason for summing the slope and asymptote, rather than treating them as individual variables?
Although we were interested to see if there was a difference, we did not have a specific prediction. Rather we tried to find a replacement for the more commonly used exponential function. We chose to sum the two because summing them leads to an Adaptation Score that represents fast and high levels of adaptation with high scores, and slow and low levels of adaptation with low scores. Anything in between can either mean an interplay between the slope and intercept. In some previous literature the focus was put on speed of

adaptation to benefit from exploration strategies. Looking at the individual slopes and asymptotes we unfortunately cannot make up any distinct effect. This might also be because we had only a few participants with higher slopes. We have added an overview of the slopes and asymptotes.

Line 214-223. After reading the comment of the other reviewer on the redundancy of the task, I understand why the authors performed this analysis, but this analysis is not clearly motivated in the paper. (I also think that in its current form this analysis will not mitigate the concern of the other reviewer, but I will leave this up to the other reviewer. I'd be happy to explain more if needed).
We have expanded our motivation on the reasons for the performed analyses.

This analysis makes me wonder about the strategies that participants used. Were most participants fairly stereotyped in where they would intercept the target? The figure showing the Mv and Ma variability correlations seems to suggest that most participants had a fairly low variance in Ma, but some participants have a rather high variance.It would also be useful to explain the relationship between Mv and Ma in more detail than mentioning that there is a correlation. Have the authors verified whether this relation is independent of target speed?
We have added a Lasso regression analysis to look at how TE was affected by different kinematic variables, among others: target speed, Mv and Ma.

Line 257. “Adaptation scores varied between -1.49 and +1.67.” Since the scores are normalized these numbers don't provide much information. It would be useful to have an overview of the (unnormalized) slopes, asymptotes and adaptation scores for all participants. It would also be useful to provide some information about the goodness of fit for the two-state lines.
See above

Figure 2 + 4. The y-axis of these figures is labelled ‘Temporal Error (ms)’. This might be confusing as it suggests that the error increases during the adaptation phase (rather than going towards zero). In addition, it seems that the numbers on the y-axis of the revised Fig 2 are seconds instead of ms.
Changed Temporal Error to Temporal Hand Error (ms) in order to clarify its meaning. Changed all TE measurements to ms.
